# New Genes Born-In or Invading Vertebrate Genomes

**DOI:** 10.3389/fcell.2021.713918

**Published:** 2021-07-06

**Authors:** Carlos Herrera-Úbeda, Jordi Garcia-Fernàndez

**Affiliations:** Department of Genetics, Microbiology and Statistics, Faculty of Biology, and Institute of Biomedicine (IBUB), University of Barcelona, Barcelona, Spain

**Keywords:** transposon-domestication, virus-invasion, Bex-Tceal, gene_birth, junk_DNA

## Abstract

Which is the origin of genes is a fundamental question in Biology, indeed a question older than the discovery of genes itself. For more than a century, it was uneven to think in origins other than duplication and divergence from a previous gene. In recent years, however, the intersection of genetics, embryonic development, and bioinformatics, has brought to light that *de novo* generation from non-genic DNA, horizontal gene transfer and, noticeably, virus and transposon invasions, have shaped current genomes, by integrating those newcomers into old gene networks, helping to shape morphological and physiological innovations. We here summarized some of the recent research in the field, mostly in the vertebrate lineage with a focus on protein-coding novelties, showing that the placenta, the adaptative immune system, or the highly developed neocortex, among other innovations, are linked to *de novo* gene creation or domestication of virus and transposons. We provocatively suggest that the high tolerance to virus infections by bats may also be related to previous virus and transposon invasions in the bat lineage.

## Introduction: Nearly Two Centuries of Honeymoons and Divorces of Scientific Fields

That Charles Darwin changed the world with his book on the origin of the species (Darwin, 1859), and quaked human thinking with its “Descent of man” (Darwin, 1871) is beyond doubt. However, he did not know what a gene was. Now, we know what a gene is, but we do not really understand where genes come from. What Darwin was aware, however, was of the relevance of Embryology and, in fact, he based his evolutionary theory in part on the old observations of embryos similarities, regardless of adult similarities, of Karl Enst von Baer (1828). In Darwin words: “community of embryonic structure reveals community of descent,” (Darwin, 1859). As a landmark example that validated the evolution/development marriage was the discovery by the Russian embryologist Kowalewski of a notochord in the urochordates (Kowalewski, 1866), noticeably even to non-specialist readers: “One could hardly open a scientific Journal or any popular essay on Natural History without meeting some allusion to the Ascidians as our ancestors” (Agassiz, 1874). Also, Haeckel and his biogenetic law (Haeckel, 1866) and his famous statement “ontogeny recapitulates phylogeny” became very popular. Therefore, at the end of the XIX century, the evolution of species was linked to developmental biology and developmental biology to cell behavior but, however, nothing about the evolution of genes, the ultimate actors of living beings.

The beginning of the XX century was dramatic for Evolution. Genes were (re)discovered, but the nascent new science, genetics, was a very disturbing third party to the evolution/development marriage and thus for comparative biology: genes were believed to have nothing to do with embryonic development (Bateson, 1922). It is true that mutation rates, allele frequencies, vast amounts of mathematics, and exquisite research in wild animal and plant population insufflate and developed a well-established evolutionary thinking, named Evolutionary Synthesis or Modern Synthesis (Huxley, 1942). Later, several Extended Synthesis that included a variety of new concepts in addition to classic Mendelian Genetics were added (Eldredge and Gould, 1972; Mayr and Provine, 1980). However, where new genes came from was not a central issue for the new or extended synthesis. Still, after Watson and Crick (1953), it was believed that genes from flies and mouse, as an example, had to be extremely different and mainly unrelated. In addition, it was thought that the human genome had to include many more genes than the fly genome, as it was obvious in terms of body plan complexity (from a human point of view).

This “old” view of evolution was shaken in 1978 by a major article that merited a Nobel prize 20 years later, the discovery of the Hox cluster (Lewis, 1978). In 1984 the homeobox was identified molecularly (McGinnis et al., 1984; Scott and Weiner, 1984) and Hox genes from mouse and human, intriguingly similar to the fly genes, discovered. The homeobox, named the “Rosetta Stone” of Biology (Garcia-Fernàndez, 2005) was the launching point of a new discipline and, indeed, the start of the reconciliation of Evolution and Embryology. With Genetics as the major actor of the so-called New-new Synthesis, New extended synthesis or Evo-Devo, the rationale was simple: if developmental genes regulate development, development regulates morphology and physiology and evolution relies on morphology and physiology, then understanding the evolution of developmental genes is the crux to understanding evolution (Baguñà and Garcia-Fernàndez, 2003). Interestingly indeed, the central period of embryonic development, the so-called phylotypic stage, was the stage where crucial (and highly conserved) developmental genes were expressed, suggesting that only earlier and final periods of development were highly evolvable. This was named the hourglass model that related development and evolution (Raff, 1996; Wagner, 2014) and the extended Hox family that is expressed there as the “zootype” (Slack et al., 1993).

In this new field, myriads of reports highlighted the conservation of genes, gene networks among large evolutionary distances and overall number of protein-coding genes in metazoans, creating a paradox at the beginning of the XX century, especially in the animal kingdom: if animals share all this, why are they so different? The solution to the paradox may well rely upon changes of gene regulation in a broad sense, from changes in *cis-*regulation, post-transcriptional regulation, or effects of gene duplication and an ever-growing list of new regulatory elements, with microRNAs, genome topology, epigenomics, long non-coding RNAs or gene editing as the latest players (Heimberg et al., 2008; Wang and Chang, 2011; Irimia et al., 2012; Cavalli and Misteli, 2013; Fromm et al., 2015; Liscovitch-Brauer et al., 2017; Deline et al., 2018). However, not much attention was given to a kind of change in genome functioning which may have an unforeseeable impact in evolution: the birth of new genes.

For half a century, most scientists believed that new protein-coding genes arise as a result of mutations in existing protein-coding genes. It was considered nearly impossible for anything as complex as a functional new protein to arise from scratch (Jacob, 1977). This was puzzling to the understanding of the evolution of metazoans, especially for those major morphological transitions that constitute evolutionary key points bound to radical innovations (Simakov and Kawashima, 2017), from the origins of multicellularity (Stanley, 1973) to the mammal placenta (Roberts et al., 2016). Fortunately, and thanks to the present affordability of new high throughput technologies, an explosion of transcriptomic and genomic data from key phylogenetic species have allowed us to widen our understanding of how such novelties have arisen (Van Oss and Carvunis, 2019).

**FIGURE 1:**
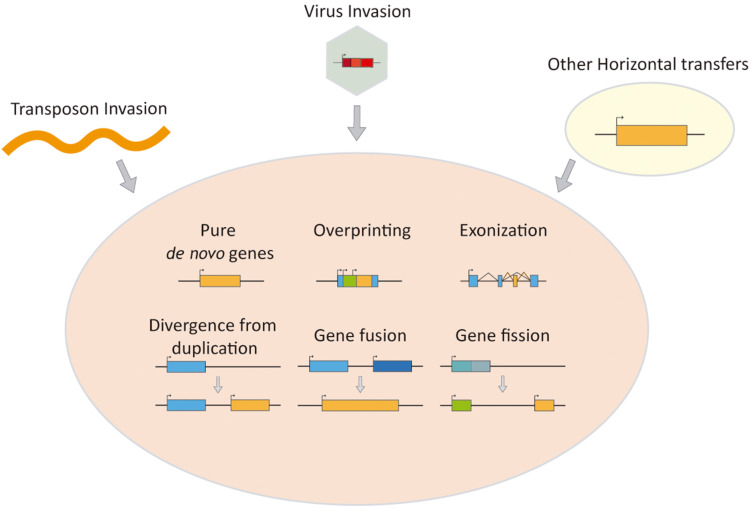
Schematics of mechanisms for generating *de novo* genes.

These disruptive novelties are often accompanied by the apparition of new genes that integrate into the current gene networks (Lavialle et al., 2013; Parrish and Tomonaga, 2018; Zhang et al., 2019; Navas-Pérez et al., 2020; Pascual-Carreras et al., 2020). These new genes (Figure 1) can of course be paralogs caused by duplication of genes already present in the genome which could allow the duplicated gene to change its sequence or its regulation (Ohno, 1970; Putnam et al., 2008; Jimenez-Delgado et al., 2009). But they can be fully *de novo* genes, which have no evident homology with any other gene within the studied species or their close relatives: the also known as orphan or taxonomically restricted genes (Singh and Syrkin Wurtele, 2020). We classify here those protein-coding new players found in old genomes from its origin and summarized the knowledge of their impact, with a special focus on its involvement in vertebrate evolution, highlighting the role of virus, virus-related elements, and transposons, when invading vertebrate and mammalian old genomes.

## Two Sides of the Same Coin: New Genes From Scratch and New Genes From Already Existing Genic Regions

**FIGURE 2:**
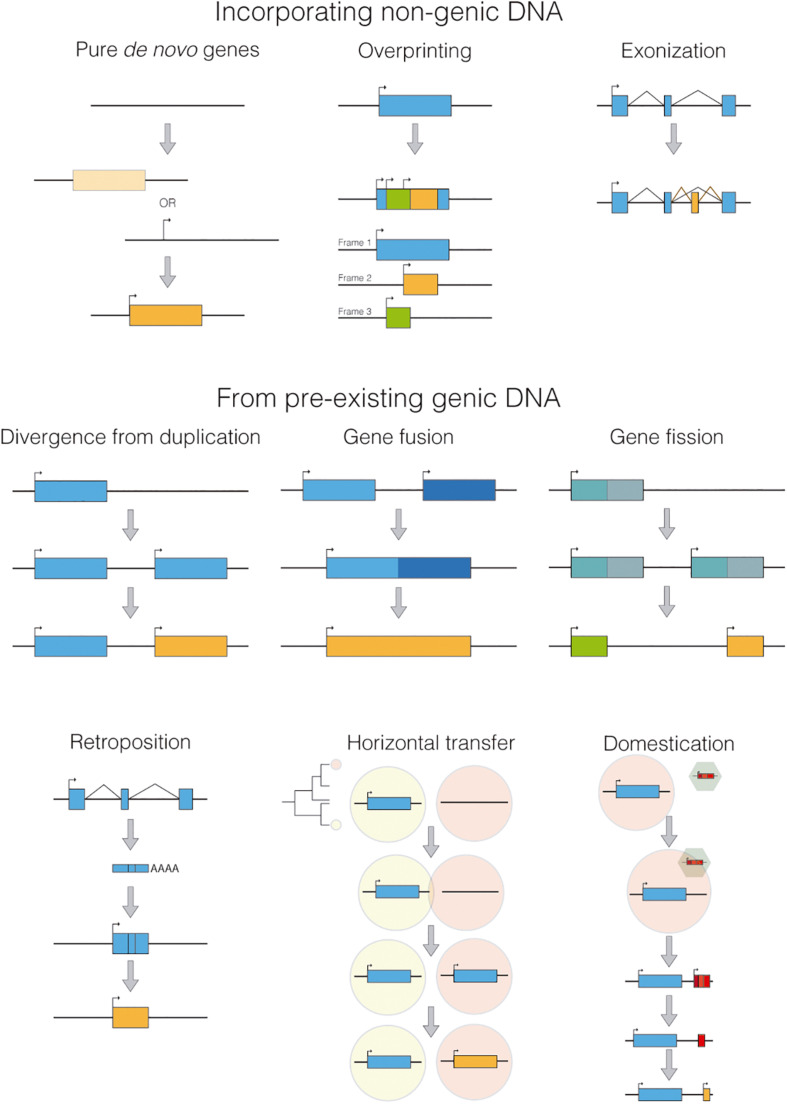
Different mechanisms for generating taxonomically restricted genes.

Although taxonomically restricted genes are a very heterogeneous group, they can be classified according to their origin (Figure 2). The true *de novo* genes emerged from ancestrally non-genic regions (wrongly called “junk DNA” in the past). These new genes can arise by mechanisms still poorly understood that involve a genomic region gaining both, transcriptional activity (maybe by transcriptional leaking) and an ORF (open reading frame) in either order (although the ORF is not necessary for non-coding genes). That would seem to be the case of the gene *blitzschnell* found in planarians (Pascual-Carreras et al., 2020) although with the current information we can not infer if the ORF originated first, or instead it was the transcriptional activity. Another mechanism that could incorporate non-genic material to the gene repertoire would be overprinting. In this process, a new ORF is created overlapping an existing one but in a different frame, resulting in two or more genes with overlapping coordinates but with substantially different amino acid sequences (Delaye et al., 2008). Finally, non-genic material can be added to an already existing gene through exonization, in which new exons are generated by random mutations in non-genic DNA, like most alternative exons regulated by NOVA (Irimia et al., 2011), a splicing factor known to be responsible for the inclusion of previously non-extant exons using cryptic splice sites near to the Nova-binding motifs. Or the well know example of the extra exon of TRPV1 (similar to the one present in Laurasiatheria mammals) that has been co-opted in vampire bats to detect hot spots on warm-blooded prey (Gracheva et al., 2011).

Meanwhile, other taxonomically restricted genes can emerge from several other mechanisms (Figure 2), such as extreme divergence from a previous duplication, gene fusion, gene fission, horizontal gene transfer, retroposition or domestication of viral or transposable elements. Remarkably, the process of domestication has been reported to be responsible for disruptive innovations, as independently evolving genes integrate into a new gene framework.

## Domesticated Retroviral Proteins Through Evolution

**FIGURE 3:**
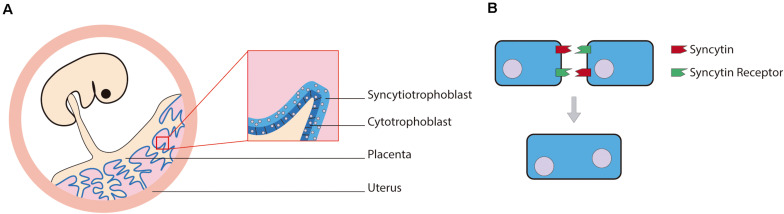
**(A)** Human example of the syncytiotrophoblast location in the placenta alongside **(B)** a diagram of the syncytium formation mediated by syncytins.

Endogenous viral elements are remnants from the integration of retroviruses into the genome and are quite abundant in the animal genomes (Katzourakis and Gifford, 2010). For example, the human genome is formed approximately by 8% of endogenous retroviral sequences (Lander et al., 2001). Throughout evolution, most of the genes from these sequences lose their function, but some of them are captured and “domesticated” in a process called exaptation. Among the examples of domesticated viral genes, the group that has most clearly influenced the evolution of mammals are syncytins (Lavialle et al., 2013). These captured viral proteins are the product of an envelope gene of retroviruses ancestrally endogenized. The envelope glycoprotein (Env) is crucial in the process of viral entry in enveloped viruses and induces fusion of the virion envelope with the cell plasmatic membrane (Sha et al., 2000). Within the human genome several Env genes can be found, but only two of them have a placental-specific expression and induce the formation of syncytia (Figure 3). Being the placenta such a defining organ in placental mammals, the syncytins responsible for its development could be expected to be orthologs in the different species, but that is not the case (Lavialle et al., 2013). Primate and mouse syncytins are not syntenic, and there is evidence pointing to independent capture events in the ancestors of each clade, as well as in the Scincidae genus Mabuya (Cornelis et al., 2017). In fact, in mammals, the different capture events can be linked with the four different main types of placental structures. In the same way, the differences between the lizard placenta present in the genus Mabuya and the mammal placenta can be traced to a completely different capture of Env genes (Cornelis et al., 2017).

Similarly, Arc genes mediate intercellular communication and synaptic plasticity via extracellular vesicles (Parrish and Tomonaga, 2018), and are homologous to the Group-specific antigen (Gag) polyproteins. In retroviruses, capsids are necessary for cellular infection and their assembly is mainly mediated by Gag (Rose et al., 2020). The similarities between Arc and Gag are not restricted to sequence, as Arc is able to spontaneously assemble into a structure that resembles to a capsid (Pastuzyn et al., 2018). In fact, Arc not only forms these capsid-like structures but also encapsulates any mRNA present during their formation. These processes allow the traffic of RNA molecules between nervous system cells (Ashley et al., 2018). Regarding the capture and evolution of this viral protein, phylogenetic analyses showed at least two independent capture events that took place in the ancestors of tetrapods and in that of schizophorans (Pastuzyn et al., 2018). In both cases, the co-option of Arc led to similar functions of RNA trafficking in the nervous system. In both lineages the closest hit was a Ty3/gypsy retrotransposon, but tetrapod Arc grouped with the fish Ty3/gypsy, while fly Arc grouped with the insect Ty3/Gypsy, indicating that while sharing significant homology with the Gag protein, it seems to have originated from the Ty3/gypsy retrotransposons from each lineage.

## Transposon-Derived Novelties in Vertebrate Evolution

As with the endogenous viral elements, transposable elements can be a source of disruptive innovations. The process of transposition can place sequences near new promoters or generate new fusion proteins. In the evolution of the adaptative immune system of vertebrates, the domestication of the RAG (recombination-activating genes) transposon was instrumental for the V(D)J recombination system, which is a process that makes possible the diversity of antibodies and T cell receptors present in the vertebrate adaptative immune system (Zhang et al., 2019). The current model, supported by the presence of ProtoRAG in the pre-vertebrate amphioxus, is that an ancestral Transib transposon with RAG1-like ORF and terminal inverted repeats, similar to the recombination signal sequences present in V(D)J, captured a RAG2-like ORF to form the ancestral RAG transposon. This event, which took place in an early deuterostome (Carmona and Schatz, 2017), was followed in jawed vertebrates by the insertion of the RAG transposon into a gene encoding an immunoglobulin-domain receptor, among other changes that suppressed RAG transposition activity to finally constitute the V(D)J recombination system.

Another example of a domesticated transposable element is the paired box (PAX) family (Paixão-Côrtes et al., 2015). These homeotic genes were discovered in 1986 (Bopp et al., 1986) and have been proved to be master regulators of the development in metazoans (Dahl et al., 1997). There are several PAX genes, and they were thought to have a monophyletic origin which was initially set at the beginning of metazoan diversification (Hoshiyama et al., 2007). However, PAX-like genes were found in protozoans placing the ancestral PAX down to the pre-metazoan era (Wang et al., 2010). The origin of the ancestral PAX gene is characterized by the domestication of a Tc1/mariner transposon, an ancient and widespread transposon family present in metazoans as well as in plants and protozoans (Garcia-Fernàndez et al., 1993). The Tc1/mariner transposase is similar to the PAX DNA-binding paired domain and its capture was probably posterior to the formation of the other two characteristics domains.

The Pax family represents not the only transcription factor formed by fusion of a transposase domain with another gene. The host-transposase fusion (HTF) genes are a group of genes that arose most probably from exon shuffling (Cosby et al., 2021), where a transposable element landed within an intron of an existing gene. Once there, the splicing machinery used the splice acceptor/donor sites pre-existing in the integrated transposon. The resemblances in the origin of several HTFs suggest then that DNA transposons are prone to be captured via alternative splicing. In fact, in tetrapod lineages, 106 distinct HTFs have been identified recently from 106 independent events (Cosby et al., 2021).

## When Can New Genes Integrate Into Existing Networks?

As we have seen, these kinds of newborn genes are not as rare as initially thought and can shape the evolution of entire groups of animals. But in order to generate a disruptive novelty, a new gene not only has to be born but to be integrated into an already existing gene network. Genes mainly involved in the phylotypic stage, the stage of development shared by all members of the phyla (Duboule, 1994) (the elongated neurula stage in the case of vertebrates) are ancient genes forming highly conserved gene networks (Irie and Sehara-Fujisawa, 2007), where the slightest variation could wreak havoc in the most crucial stages of development. Oppositely, gene networks acting in very early or very late stages of development tend to show more variation and have less conserved or even new genes implicated (Irie and Kuratani, 2011). In this context, the “hourglass model” (Drost et al., 2017), recently also found at the gene regulatory level (Liu et al., 2020), suggest that if a new gene is born, it may preferentially end up functioning at very early (e.g., placenta) or very late stages of the life cycle (e.g., adaptative immunity, synaptic plasticity, body size regulation).

A good example besides the ones already mentioned would be the aforementioned taxonomically restricted gene family, blitzschnell (Pascual-Carreras et al., 2020). Found only within the order Tricladida (planarians), this family is composed of 11 genes and four pseudogenes. It can be further divided into five subfamilies, with one of them organized in a cluster formed by tandem duplication events. Three of the subfamilies are coding and have been reported to regulate the growth/degrowth according to nutrient intake. Thus, these *de novo* genes have been integrated into an evolutionary conserved metabolic network, the insulin/Akt/mTOR network responsible for growth (Saxton and Sabatini, 2017) and other mechanisms at a late stage of the life cycle.

## A Whole New Cluster From a Domesticated Transposable Element: Impact Into the Evolution of the Eutherian Brain

**FIGURE 4:**
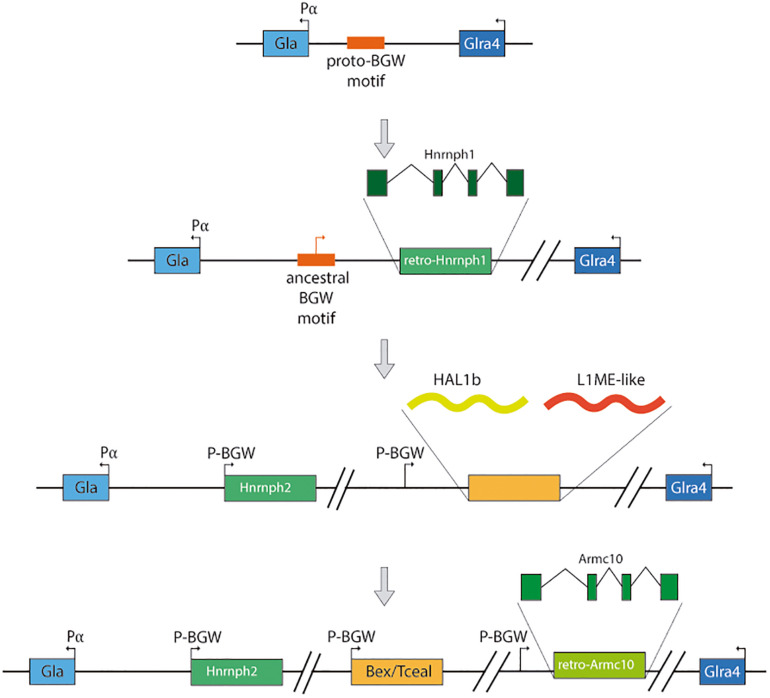
Proposed steps for the Bex/Tceal cluster formation.

Recently Navas-Pérez et al. (2020) presented evidence of the expansion of a domestication event into the Bex/Tceal multigenic family, constituting a cluster of 14 genes in the X chromosome of the ancestor of eutherians, after the divergence of the marsupial-placental clades. The domestication event proposed (Figure 4) consists of six steps: (i) A proto-BGW motif (Winter and Ponting, 2005) existed upstream of the alpha-galactosidase (Gla) promoter (P α) in the X chromosome of the ancestor of eutherians and metatherians; (ii) In the eutherian lineage, a retrotranscribed Hnrnph1 was inserted next to the BGW motif and upstream of Gla, creating the Hnrnph2 retrogene; (iii) The region containing the co-opted BGW motif and Hnrnph2 suffered a duplication and HAL1b and L1ME-like retrotransposons were inserted in the vicinity; (iv) The BGW motif and the ORF created by the insertion of the retrotransposons conformed the proto-Bex/Tceal with the YY1 binding site from HAL1b preserved; (v) The BGW motif and the YY1 binding site of a Bex/Tceal gene duplicated upstream of a retrocopy of the Armc10 gene, giving rise to the ArmcX ancestral gene; and (vi) The Bex/Tceal and ArmcX gene families expanded forming the BGW cluster before the diversification of the placental lineage.

Regarding their expression, according to the available data from adult organs, most genes present a tissue-enriched pattern, with the brain be the organ showing the highest expression levels in most of the paralogs. Navas-Pérez et al. (2020) also reported an analysis of the expression via *in situ* hybridization during mouse development, where they observed that especially the Bex genes were highly and widely expressed with Bex3 strongly expressed in the nervous system.

Functional analyses of Bex/Tceal genes have been performed *in vitro*, where Bex3 for example was linked to neuronal physiology, and also through mutant mouse lines. The homozygous mutant lines showed subtle facial differences to the naked eye due to cranial morphology aberrances. The cerebellum and brain showed a reduction in size a fact that may be linked to the behavioral defects observed in the mutant mice. Particularly, the mutants showed impairment in social interactions, nest building, working memory, and object recognition memory. This mutant phenotype coupled with the reported physical interaction of BEX3 with TSC1 (Yasui et al., 2007) could mean that Bex3 is preventing the TSC1/2 complex from interacting with mTORC2, inhibiting this pathway. Thus, Bex3 would be fine-tuning the regulation of the mTOR pathway and its deregulation was suggested to be related to autism spectrum disorder in humans (Ganesan et al., 2019).

Some of the structural features present in BEX and TCEAL families have been detected in the ancestral transposon HAL1b (Navas-Pérez et al., 2020), which indicates that they were preserved along the domestication process., and a positive selection signature can be found in particular cases, suggesting than ancestral genes went through an adaptative process before the diversification of placental mammals. The differences in the neocortex complexity between eutherian and non-eutherian mammals (Cheung et al., 2010) may thus be linked to genomic novelties that emerged during this transition affecting neural development, and maybe, to the appearance of the Bex/Tceal cluster.

The ability to affect neural proliferation, however, seems to have been acquired at some point during the formation of the cluster and not be intrinsic to the eutherian Bex/Tceal ancestor. This was proved electroporating a synthetically reconstructed version of the ancestral Bex/Tceal protogene, as well as the murine Bex3 and Tceal7 into the neural tube of chicken embryos to serve as a non-eutherian vertebrate environment. Expression of Bex3 and Tceal7 generated a noticeable increase in cell proliferation in the embryonic neural tube, similar to what had been reported in mammalian cell cultures (Calvo et al., 2015). Meanwhile, the reconstructed protogene was not able to trigger cellular proliferation, which could mean that this ability was gained afterward its formation.

## Final Remarks: New Genes, Old Genomes, Innovations, Adaptations And, Maybe, Viral Fighting Solutions

Here, we have reviewed a series of cases in which new born genes have been instrumental for the emergence of novelties, some of them shaping a whole taxon. The *de novo* genes, irrespectively if they have been gained by horizontal transfer event or have been formed within the genome, are an engine of evolution, providing new tools for the regulatory networks of extant genomes. Therefore, disruptive novelties may emerge when new genes integrate into old genomes. In the particular case of vertebrates, it is clear that new exons and whole genes were born by recruitment of non-coding DNA and domestication of transposons and virus, that continuously invaded the genome trough vertebrate evolution. Particularly noticeable are some periods linked to the appearance of remarkable innovations, such as the origin of the adaptative immune system, the origin of the placenta, or the deployment of a well-developed neural cortex. Further, in the time of COVID pandemics by SARS-CoV-2, it is tempting to speculate, and timely to investigate, if one of the most successful lineages of mammals, the Chiroptera, is particularly remarkable with regards to *de novo* genes. Bats are particularly more tolerant to viral infections than most mammals, including humans. As somewhat expected for their high resistance to viral infections, the bat genome shows gene expansions and deletions related to the immune system gene network (Jebb et al., 2020) but intriguingly also increasing numbers and high diversity of endogenous viral elements^1^ and extremely variable numbers and types of transposon remnants, often bat family or bat species-specific (Jebb et al., 2020). All this together is suggestive of a high level of recent events of virus and transposon invasions in the bat lineage. Whether those invasions helped, in fact, the deployment of virus resistance, in an unexplored but exciting similarity to the original function of the CRISPR/Cas9 system in bacteria, the primitive procaryotic acquired adaptative immunity system, is something that remains to be investigated, with the ambition to learn how to fight, in humans, virus related diseases.

## Author Contributions

CH-Ú and JG-F: conceptualization, writing, review, and editing. JG-F funding acquisition. Both authors contributed to the article and approved the submitted version.

## Conflict of Interest

The authors declare that the research was conducted in the absence of any commercial or financial relationships that could be construed as a potential conflict of interest.

## Footnotes

1 https://genome-public.pks.mpg.de
